# The Relationship Between Green Space and Prosocial Behaviour Among Children and Adolescents: A Systematic Review

**DOI:** 10.3389/fpsyg.2020.00859

**Published:** 2020-04-30

**Authors:** I Gusti Ngurah Edi Putra, Thomas Astell-Burt, Dylan P. Cliff, Stewart A. Vella, Eme Eseme John, Xiaoqi Feng

**Affiliations:** ^1^Population Wellbeing and Environment Research Lab (PowerLab), School of Health and Society, Faculty of Social Sciences, University of Wollongong, Wollongong, NSW, Australia; ^2^Menzies Centre for Health Policy, University of Sydney, Sydney, NSW, Australia; ^3^National Institute for Environmental Health, Chinese Center for Disease Control and Prevention, Beijing, China; ^4^School of Public Health, Peking Union Medical College, The Chinese Academy for Medical Sciences and Tsinghua University, Beijing, China; ^5^School of Education, Early Start, Faculty of Social Sciences, University of Wollongong, Wollongong, NSW, Australia; ^6^Illawarra Health and Medical Research Institute, University of Wollongong, Wollongong, NSW, Australia; ^7^School of Psychology, Faculty of Social Sciences, University of Wollongong, Wollongong, NSW, Australia; ^8^School of Public Health and Community Medicine, University of New South Wales, Sydney, NSW, Australia

**Keywords:** prosociality, altruism, nature, environment, green space quantity, green space quality, children, adolescents

## Abstract

The plausible role of nearby green space in influencing prosocial behaviour among children and adolescents has been studied recently. However, no review has been conducted of the evidence testing the association between green space and prosocial behaviour. This systematic review addresses this gap among children and adolescents. Within this review, we propose a conceptual framework describing potential pathways linking green space to prosocial behaviour, discuss the direction, magnitude, moderators, and mediators of the association, and develop a narrative synthesis of future study directions. Out of 63 extracted associations from 15 studies, 44 were in the positive or expected direction, of which 18 were reported to be statistically significant (*p* < 0.05). Overall, the current evidence shows that exposure to green space may potentially increase prosocial behaviour among children and adolescents, with some contingencies (e.g., child's sex and ethnic background). However, the volume and quality of this evidence is not yet sufficient to draw conclusions on causality. Further, heterogeneity in the indicators of green space exposure could lead to mixed findings. In addition, none of the included studies investigated potential mediators. Nevertheless, this review provides preliminary evidence and a basis for further investigation with rigorous study methodology capable of drawing causal inferences and testing potential effect modifiers, linking pathways, and relevant green space measures.

## Introduction

Prosocial behaviour is increasingly recognised as an important part of child development (Dunfield, 2014). It includes a range of behaviours that “benefit others or at very least promote harmonious relations with others” (Hay, 1994, p. 33). Prosociality among children is characterised by the presence of positive interactions, such as sharing, helping, cooperating, and comforting (Hay, 1994; Dunfield, 2014; Hammond et al., 2015; Piotrowski et al., 2015; Wittek and Bekkers, 2015). Prosocial behaviour emerges in early childhood and can progressively increase in variety, frequency, and complexity as children get older (Hay et al., 2004; Knafo et al., 2008; Brownell, 2013). In addition, newly established social networks (e.g., friendship) and the growth of socio-cognitive capabilities potentially lead to more opportunities for older children to behave prosocially (Hay and Cook, 2007; Abrams et al., 2015; Eisenberg et al., 2015). However, the evidence suggests that prosocial behaviour might decline in early- and middle-adolescence, but may start to rebound in late adolescence or early adulthood (Eisenberg et al., 2015).

A current body of literature highlights the importance of prosocial behaviour in positively contributing to aspects of youth development. Positive outcomes include greater academic success (Collie et al., 2018; Gerbino et al., 2018), social competence (Bar-Tal, 1982), and problem-solving skills (Carlo et al., 2012; Eisenberg et al., 2015). Prosocial behaviour is considered a psychosocial asset (Leventhal et al., 2015), that contributes to better quality peer relationships (Caputi et al., 2012), lower reported aggression (Swit, 2012; Obsuth et al., 2015), and favourable subjective well-being (Aknin et al., 2012, 2015; Proctor and Linley, 2014; Yang et al., 2019). Previous work also suggests that prosocial behaviour was associated with child health-related outcomes and behaviours including fewer externalising and internalising behavioural problems (Flynn et al., 2015; Flouri and Sarmadi, 2016), lower screen time (Healy and Garcia, 2019), and optimal cardiometabolic health (Qureshi et al., 2019). Given these potential benefits for positive health, psychological, and social aspects, promoting prosocial behaviour development beginning in early childhood is important.

The development of prosocial behaviours is jointly determined by factors that can be broadly described as personal and environmental characteristics (Piliavin, 2001). Genetic factors (Fortuna and Knafo, 2014; Israel et al., 2015; Knafo-Noam et al., 2015), gender (Abdi, 2010; Kok et al., 2018), personality traits or self-concepts (Cauley and Tyler, 1989; Gallitto and Leth-Steensen, 2019), and empathy (Garaigordobil, 2009; Williams et al., 2014) are the factors that contribute to prosocial behaviour differences between individuals. In addition, published literature has also suggested that cultural background and values are correlates of prosocial behaviour (Richman et al., 1988; Smith et al., 2019). Socio-environmental factors such as parental influences (parental nurturing, parent-child relationship, parental warmth, parental socialisation; Carlo et al., 2010; Pettygrove et al., 2013; Ferreira et al., 2016; Pastorelli et al., 2016) and peer influences (Fujisawa et al., 2008; Fabes et al., 2012; Lai et al., 2015; Lee et al., 2016; Oldfield et al., 2016; Silke et al., 2018) are important predictors for the development of prosocial skills among children and adolescents. Moreover, the exposures to prosocial content from media positively influence prosocial acts, whereas the use of violent media exhibits negative associations (Bar-on, 2000; Greitemeyer, 2011; Prot et al., 2014; de Leeuw et al., 2015). Aspects of the physical environment such as schools are also important to promote prosocial behaviour since schools enable social interactions among children and adolescents through organised cooperative learning activities in class, and through opportunities for play (Wentzel, 2015). The presence of other physical environments that facilitate social contacts and interactions such as green space in urban environments potentially serves as an additional space for children to develop and practice prosocial acts.

Green spaces are public areas that include natural vegetation components, such as grass, trees, and/or shrubs that people commonly utilise as gathering places for recreation, sport, relaxation, and other social activities (Dinnie et al., 2013; Dennis and James, 2016; Jennings and Bamkole, 2019). Those areas can be naturally created, such as forests, other landscapes with natural entities or human-made or built environments that contain natural vegetation, such as gardens and parks (Hartig et al., 2014; Taylor and Hochuli, 2017). While children in urban areas tend to spend less time in outdoor activities and have less social contact with other children (Singer et al., 2009), the presence of nearby green space might promote positive social interactions that lead to prosocial behaviour development. The plausible influence of urban green space on child prosocial acts is increasingly being studied in recent years (Amoly et al., 2014; Balseviciene et al., 2014; Richardson et al., 2017; McEachan et al., 2018; Whitten et al., 2018; Andrusaityte et al., 2019). However, no systematic review of these studies is available so far.

This systematic review aimed to evaluate the available literature on the association between urban green space and prosocial behaviour among children (0–12 years) and adolescents (13–18 years). These age ranges were selected based on a previous systematic review on prosocial behaviour among adolescents (Silke et al., 2018). Further, we propose a conceptual framework and provide discussion of the potential mechanisms linking green space and prosociality. In addition, a narrative synthesis of the existing published literature on green space and prosocial behaviour nexus is presented, followed by the discussion of our findings and future study directions.

## Potential Mechanisms Linking Green Space and Prosocial Behaviour

Health benefits due to neighbourhood green space exposures in urban environments have been well-documented among children that include better mental health and well-being (Flouri et al., 2014; Feng and Astell-Burt, 2017c,d; McCormick, 2017; Vanaken and Danckaerts, 2018), more physically active and/or less screen time (Roemmich et al., 2006; Sanders et al., 2015; Akpinar, 2017), and reduced odds of respiratory health problems (Feng and Astell-Burt, 2017b; Tischer et al., 2017; Eldeirawi et al., 2019). Moreover, favourable health outcomes due to green space exposure across the lifespan have been reported in some recent systematic reviews (Lee and Maheswaran, 2011; van den Berg et al., 2015; Kondo et al., 2018; Twohig-Bennett and Jones, 2018). However, the potential association between greenness and prosocial behaviour and its underlying mechanisms have not been widely reported.

**Figure 1:**
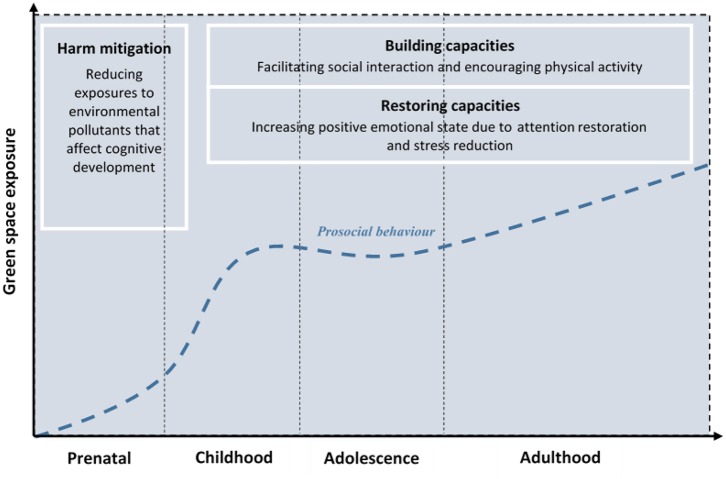
Potential pathways linking green space to prosocial behaviour. Adapted from Markevych et al. (2017) and Ben-Shlomo et al. (2014).

Scholars in multidisciplinary fields suggested a conceptual model to help understand the mechanisms linking urban green space to health outcomes. Three domain pathways are proposed and these include (i) harm mitigation (e.g., reducing harmful environmental exposures—air pollution, noise, heat), (ii) restoring capacities (e.g., restorative effects, stress recovery), and (iii) building capacities (e.g., promoting physical activity, facilitating social cohesion; Markevych et al., 2017). Under the frame of this theoretical model, we elaborated potential mechanisms linking urban green space to prosocial behaviour. In addition, we also adopted the concept of life course epidemiology which suggests that exposures to physical or social factors during the life course might have long term effects on later disease risk or health outcomes (Kuh et al., 2003; Ben-Shlomo et al., 2014). Based on this concept, we identified potential critical and sensitive periods for the influence of green space on the development of prosocial behaviour. Our combined model is shown in Figure 1 and discussed below.

Harm mitigation may be the first pathway linking green space to child prosocial behaviour. Exposures to environmental pollutants during vulnerable windows, such as prenatal or early postnatal periods might have adverse impacts on child cognitive development (Dadvand et al., 2015), which in turn, influences prosocial behaviour. Ren et al. (2019) conducted a cross-sectional study to examine the associations of prenatal exposure to outdoor air pollution on prosocial behaviour among China's pre-schoolers. Exposures to PM_10_ (particulate matter < 10 μm in diameter) and PM_2.5_ (particulate matter < 2.5 μm in diameter) during the full gestation period were reported to be associated with increased odds of abnormal range of prosocial behaviour after controlling for child-related factors, maternal factors, and socio-economic status. Meanwhile, past work suggested that air-related pollution can be reduced by the presence of green space (Su et al., 2011; Dadvand et al., 2012a,b). Previous studies also found the association between urban greenness and cognitive development among children was partly explained by reduction in air-related pollution (Dadvand et al., 2015; Liao et al., 2019). Therefore, early and frequent exposures to nearby greenness can positively affect later prosocial behaviour by mitigating harmful environmental stressors during windows of susceptibility such as during the prenatal period. Furthermore, negative effects of prenatal exposure to air pollution on prosociality can be attenuated by factors driving cognitive development, such as learning activities and social interactions that can occur in other settings (e.g., schools; Weinstein and Bearison, 1985; Gustin et al., 2018).

Childhood could be one of the critical periods for the green space-prosociality association. Critical period refers to a specific time window in which exposure has effects on the development and subsequent outcome (Kuh et al., 2003). While prosocial behaviour can progressively increase with age during childhood, exposures to green space might help to elevate prosocial behaviour development through mechanisms of building and restoring capacities. Moreover, late childhood can be considered as the sensitive period for the association between green space and prosociality due to exposures to green space might have a greater effect than it would be at other childhood periods. Older children widen their friendships and develop socio-cognitive skills (Hay and Cook, 2007; Abrams et al., 2015; Eisenberg et al., 2015). They tend to have more social interactions and behave more prosocially than their younger counterparts and the presence of nearby green space might multiply these opportunities. According to the building capacities pathway, green space provides attractive places for children to foster social interactions and then facilitate prosocial behaviour development. This is supported by the social network theory which posits that repeated and frequent interaction among individuals brings opportunities for cooperation and helps to build trustworthiness, which in turn, stimulates individuals to perform prosocial behaviour toward others (Wittek and Bekkers, 2015). In addition, the intergroup contact hypothesis contends that time spent interacting with people from different backgrounds can promote positive intergroup attitudes and decrease prejudice (Allport et al., 1954; Davies et al., 2011). A study conducted by Meleady and Seger (2016) showed that imagining social interactions with outgroup members can encourage prosocial behaviour and the association is mediated by increased trust. Furthermore, some previous studies suggested that green space potentially facilitates social interactions among adults (Kazmierczak, 2013; Hong et al., 2018; Aram et al., 2019; Jennings and Bamkole, 2019). These studies indicate that green space can possibly influence prosocial behaviour through increased social interactions that align with the nature of prosociality which is developed and practised through frequent interaction (Oerlemans et al., 2018). Neighbourhood green space also can attract children to engage in outdoor physical activity with peers (Sanders et al., 2015; Ward et al., 2016), which in turn brings opportunities to foster prosocial behaviour (Di Bartolomeo and Papa, 2017).

Other theoretical perspectives help explain the possible roles of green space for restoring capacities in relation to prosocial behaviour. According to Ulrich's psycho-evolutionary theory (PET), natural environments are best suited for humans as places where we initially evolved and humankind's survival was reliant on nature before the agricultural revolution. Emotional responses upon natural environments are viewed as part of feeling connected to nature and as being “central to the psychological components of stress and restoration” (Ulrich et al., 1991, p. 207). PET is more commonly known as stress reduction theory (SRT) which suggests that contact with natural environments can reduce the levels of stress (Ulrich, 1983). Another complementary theory, attention restoration theory (ART) contends that taking time in natural environments reduces attention-demanding tasks and allows to restore attention thereby building more positive emotional and psychological responses (Kaplan, 1995; Ohly et al., 2016). Zhang et al. (2014) reported that positive emotions mediate the association between exposures to greenery perceived as beautiful and prosocial behaviour among adults. Positive emotional states due to exposures to nature can lead to greater prosocial tendencies by changing from an individual to collective mental frame (Schwartz et al., 2019). In addition, Goldy and Piff (2020) argued that contact with natural environment can increase attention to others and enhance prosocial behaviour through psychological processes of positive feelings that include feelings of awe and perception of beauty.

Building and restoring capacities might interact to link green space and prosocial behaviour among children and adolescents. For example, children who spend time in green space for having friendly talks and plays with friends may also experience attention restoration due to viewing natural vegetation. Frequent exposure to green space may be required to enable repeated and increased social interactions, as well as to build positive emotionality, that in turn facilitate prosocial behaviour development. Early and longer accumulation of exposure to green space may generate greater levels of benefit for prosocial behaviour, particularly in childhood as critical periods and late childhood as the sensitive period. However, the increase of prosocial behaviour associated with accumulated green space exposures in adolescence might not be as high as in childhood since the natural decline of prosociality is reported in this period (Eisenberg et al., 2015). Another possible scenario is that accumulated exposures are insufficient to lessen or moderate the intrinsically-caused decline in prosocial behaviour. Later, prosocial behaviour may start to rebound in early adulthood (Eisenberg et al., 2015) and the accumulation of exposure to green space may help to increase the levels of prosocial behaviour.

Having outlined a model by which green space may influence the development of prosocial behaviour across childhood and adolescence, the remainder of this paper is dedicated to a systematic review of existing literature to examine how the published evidence addresses the hypothesised direction and magnitude of association, potential mediators, moderators, and temporal nature.

## Methods

### Search Strategy and Selection Criteria

This review was conducted following the guidelines from the Preferred Reporting Items for Systematic reviews and Meta-Analysis (PRISMA; Moher et al., 2009). The literature search was carried out in 5–6 October 2019 using nine frequently used databases, including PubMED (US National Library of Medicine, Maryland, U.S.), Scopus, ScienceDirect (Elsevier, Amsterdam, Netherlands), Web of Science (Clarivate Analytics, Philadelphia, U.S.), PsycINFO, PsyschARTICLES (American Psychologist Association, Washington D.C., U.S.), CINAHL (EBSCO Publishing, Massachusetts, U.S.), Cochrane Library (John Wiley & Sons, New Jersey, U.S.), and ProQuest (ProQuest LLC, Michigan, U.S.). Guidance on the search terms selected was obtained from recently published systematic reviews on green space (Houlden et al., 2018; Vanaken and Danckaerts, 2018) and prosocial behaviour (Oviedo, 2016; Silke et al., 2018; Vilar et al., 2019). The terms as presented in Table 1 were searched in the titles, abstracts, and/or keywords of the articles. In addition, references from eligible articles were also searched.

**Table 1:** Search terms and strategy used to search relevant literature.

**Main keywords**	**Search terms**
Green space	“green space” OR greenspace OR greenness OR greenery OR green OR “green area” OR landscape OR wilderness OR wild OR natur^*^ OR park OR garden OR playground OR playspace OR “play space” OR “open space” OR recreation OR vegetation OR wood OR woodland OR tree OR plant OR grass OR forest OR shinrin-yoku
Prosocial behaviour	prosocial^*^ OR pro-social^*^ OR altruis^*^

* *Truncation symbol used to enable search all possible variations of the word*.

### Eligibility Criteria

The inclusion criteria consisted of studies that; (1) were peer-reviewed research articles, (2) had quantitative observational or experimental design; (3) investigated association between green space as an exposure that includes objective and/or subjective measures (quantity, quality, or both) and prosocial behaviour as either an outcome or as a mediator of a health outcome; (4) were published in English; and (5) included participants ≤ 18 years of age. No restriction on publication date was applied. Published articles that only contained an abstract (e.g., conference proceedings) were excluded.

Prosocial behaviour among children and adolescents was the outcome of interest. In this review, prosociality was defined as a range of positive behaviours that include offering help, sharing, cooperating, and comforting. The outcome focuses on the behavioural aspect rather than cognitive or affective responses (e.g., kindness, love, etc.). Meanwhile, green space refers to naturally-created areas or built environments that bear natural vegetation. Green space exposure in this review considered all characteristics of green space in accordance with the keywords provided (presented in Table 1). Green space characteristics measured using land cover maps, remote sensing data, physical observation, and audits were categorised as objective measures, whilst green space exposure data collected through interviews and questionnaires were assigned as subjective measures (Houlden et al., 2018; Vanaken and Danckaerts, 2018). Green space measures can also be classified as assessing quantity which refers to amount of green space available locally within a particular administrative area (e.g., average greenness, percentage of green space), while quality of green space is evaluated by some aspects that influence the usability (e.g., cosiness, safety, amenities, facilities, attractiveness, etc.; McCormack et al., 2010; Marselle et al., 2014; Feng and Astell-Burt, 2017d, 2018). In addition, studies examining subjective connectedness to nature were also taken into account following a previous systematic review on green space (Houlden et al., 2018).

### Selection Strategy and Data Collection

All articles retrieved using the search terms in the selected databases were downloaded into EndNote. Duplicate articles were removed either using the EndNote function or manually. Two reviewers (IP and EJ) independently assessed the title and abstract of the published articles using the same inclusion criteria, followed by the full-text assessment. Further, any discrepancies between the two reviewers were discussed and consulted with a third reviewer (TA). Information about publication details, study design, sample size, participant characteristics, exposure concept and measurement, measure instrument of prosocial behaviour, and the results were extracted into Table 2.

**Table 2:** Summary of study characteristics and results.

**References, country**	**Study design**	**Sample size (age)**	**Green space exposure concept**	**Green space data source**	**Prosocial behaviour measure**	**Confounders adjusted in the model**	**Methods**	**Results in adjusted model**	**Quality**
Amoly et al. (2014), Spain	Cross-sectional study	2,111 (7–10 years)	a. Time spent playing in green spaces *(a total number of hours during the last school period and summer holidays)*; b. Residential surrounding greenness in buffers of 100, 250, and 500 m; c. School greenness in a buffer of 100 m; d. Home-school greenness *(average residential and school surrounding greenness in a buffer of 100 m, weighted by daily time spent at home and school)*; e. Residential proximity to a major green space *(a binary variable indicating whether the child's home within 300 m of a major green space)*.	Questionnaires; NDVI	Parent-reported prosocial scale from SDQ (*a continuous variable)*.	Child's sex, school level, ethnicity, preterm birth, breastfeeding, exposure to environmental tobacco smoke, maternal smoking during pregnancy, responding person, parental educational achievement, parental employment status, and neighbourhood socioeconomic.	Quasi-Poisson mixed-effects models	No statistically significant association was found between all green space indicators and prosocial behaviour (*non-significant in expected direction)*.	Fair
Andrusaityte et al. (2019), Lithuania	Cross-sectional study.	1,489 (4–6 years)	a. Time spent in a city park (*hours per week)*; b. Residential surrounding greenness in buffers of 100 m.	Questionnaires; NDVI	Parent-reported prosocial scale from SDQ (*a binary outcome: borderline/abnormal* vs. *normal)*.	Child's sex, birth weight, wheeze, asthma, allergy, BMI, breastfeeding, siblings, paracetamol and antibiotic usage during the first year of life, maternal education, tobacco smoke, age at childbirth.	Logistic regression	Increased time spent in city parks per 1 h per week was associated with decreased odds of borderline/abnormal prosocial behaviour: aOR = 0.98 (0.96, 0.99) (*significant in expected direction*). Non-significant association was found for residential surrounding greenness (*non-significant in expected direction)*.	Fair
Balseviciene et al. (2014), Lithuania	Cross-sectional study.	1,468 (4–6 years)	a. Residential surrounding greenness in a buffer of 300 m; b. Proximity to the nearest city parks *(transformed using the square root function in meters)*.	NDVI	Parent-reported prosocial scale from SDQ (*a continuous variable)*.	Child's age, sex, and parenting stress.	Linear regression	Analysis was stratified by mother's educational level. Increased distance to city parks was negatively associated with prosocial behaviour among lower education group: β = −0.029 (*p* < 0.05) *(significant in expected direction)*. Residential greenness was negatively associated with prosocial behaviour among higher education group: β = −1.104 (*p* < 0.05) *(significant in unexpected direction)*.	Fair
Bates et al. (2018), USA	Experimental study (one- group post-test-only design)	3,345 and 3,710 observations at the first (T1) and second (T2) time, respectively (age ranges from pre-kindergarten to 8th grade)	Schoolyard renovation by increasing the presence of natural components (e.g., grass, trees) and also the quality (e.g., aesthetics; facilities).	In-person observation	Positive social interaction, measured by behavioural mapping using System for Observing Children's Activity and Relationship during Play (SOCARP). It was measured two times (T1, T2) after schoolyard renovation.	No confounders adjusted in the analysis	Chi-square test	The percentage of observed positive social interaction or prosocial behaviour increased from T1 (27.10%) to T2 (35.20%) (*p* < 0.001) *(significant in expected direction)*.	Poor (no pretest, no randomisation)
Carrus et al. (2015), Italy	Experimental study (two- group post-test-only design)	39 (1.5–3 years)	Children's spending time in school green space vs. in internal space of school	In-person observation	Positive social interaction, measured by a behavioural checklist to record frequency of positive relational behaviours	No confounders adjusted in the analysis	ANOVA	After children were exposed to green space, more frequent positive relational behaviours were observed on days when children spent time in school green space compared to days when they did not (*p* = 0.038) *(significant in expected direction)*.	Poor (no pretest, no randomisation)
Dopko et al. (2019), Canada	Experimental study (two- group post-test-only design)	80 (mean age = 10.49 years)	Children' spending time outdoors at the nature school vs. indoors at the museum	In-person observation	Using two tasks: a. A windfall task by asking children to imagine that they received money and what they decided on four available options (buy things they want, give to charity, spend on gifts for other people, and save for the future). Children who decided for charity and spending on gifts for other people represent higher prosociality. b. A tangram task by asking children to imagine that they assigned 11 tangrams from three categories: easy, medium, and hard to someone else in their class. Children who assigned more tangrams in easy and medium categories, and few in hard category represent higher prosociality.	No confounders adjusted in the analysis	Paired sample *t*-test	Windfall task: Mean score for spending money on charity was statistically higher among children visiting nature school than museum: β = 3.66 (0.06, 7.26) *(significant in expected direction)*. Mean score for spending money on gift was lower among children visiting nature school than museum: β = −4.15 (−8.32, 0.03) *(non-significant in unexpected direction)*. Tangram task: Mean score for assigning easy tangram was statistically higher among children visiting nature school than museum: β = 0.74 (0.01, 1.46) *(significant in expected direction)*. Mean score for assigning hard tangram was statistically lower among children visiting nature school than museum: β = −1.29 (−2.15, −0.42) *(significant in expected direction)*.	Poor (no pretest, no randomisation)
Mayfield et al. (2017), USA	Experimental study (two- group pretest-post-test design)	Two elementary schools for each intervention and control groups. This study included 3,588 SOCARP scans representing 1,196 child recess days with 3 rotation conducted.	The intervention was carried out by improving the quality of playground through adding playground marking with colourful interactive games. In addition, intervention schools received equipment to use with the game and training sessions for teachers.	In-person observation	Positive social interaction, measured by behavioural mapping using System for Observing Children's Activity and Relationship during Play (SOCARP).	Scans nested within days nested with schools	Mixed- effects regression analysis	There was a non-significant decrease in prosocial behaviour in the verbal or physical manner before and after the intervention (*non-significant in unexpected direction*).	Fair
McEachan et al. (2018), UK	Longitudinal study	2,594 (aged 0 at baseline, 4 years at follow up)	a. Satisfaction with green space *(asked among a sub-sample of 832 (32%) only)* b. Time spent playing outside *(minutes per week calculated for winter and summer months - asked among a sub-sample of 832 (32%) only)* c. Residential surrounding greenness in buffers of 100 m, 300 m, and 500 m	Questionnaires; NDVI	Parent-reported prosocial scale from SDQ (*a continuous variable)*	Child's age, sex, maternal age, cohabitation status, maternal education, subjective poverty, household size, neighbourhood deprivation index, mother's smoking behaviour, and mother's treatment record of mental disorder	Linear regression	Analysis was stratified by ethnicity (white British vs. south Asian). Satisfaction with green space was significantly associated prosocial behaviour among south Asian children only: β = 0.20 (0.02, 0.38) *(significant in expected direction)*. Time spent playing outside was not associated with prosocial behaviour among both ethnicities *(non-significant in expected direction for south Asian children and non-significant in non-reported direction for white British children)*. Residential greenness in all buffer distances were not associated with prosocial behaviour among both ethnicities *(non-significant in expected direction)*.	Good
Odgers et al. (2012), UK	Cross-sectional study	2,024 (12 years)	Percentage of green space in a buffer of 0.5 mile (*measured only among a sub-sample of 200 neighbourhoods*)	A systematic social observation using Google Street view	A combined parent and teacher's reports of Revised Rutter Parent Scale for School-Age Children (*a continuous variable)*	No confounders adjusted in the analysis	Linear regression	No association was observed between percentage of green space and prosocial behaviour (*non-significant in unexpected direction*).	Poor (no control for confounders)
Park et al. (2016), South Korea	Experimental study (one- group pretest-post-test design)	336 (5–7 years)	Participation in 24-session horticultural activity program that included indoor and outdoor activities, such as transplanting, planting seeds, making and applying eco-friendly fertilizer, observing vegetable plants, harvesting, etc.	In-person observation	Teacher-reported of prosocial behaviour using the revised questionnaire with four subscales (helping, sharing, cooperation, kindness) (*a continuous variable)*	No confounders adjusted in the analysis	Paired sample *t*-test	All prosocial behaviour scales (helping, sharing, cooperation, kindness) increased from pretest to post-test *(significant in expected direction)*.	Fair
Richardson et al. (2017), UK	Longitudinal study	2,909 (aged 4 years at baseline, 6 years at follow-up)	a. Percentage of park space in a buffer of 500 m b. Percentage of total natural space in a buffer of 500 m c. Garden access (*indicating whether the child had access to a private garden*)	Land cover map; Questionnaire	Parent-reported prosocial scale from SDQ (*a continuous variable)*	Child's age, sex, screen time, household income, educational attainment, carer's mental health, and neighbourhood socio-economic status	Linear regression	Analysis was stratified by the child's sex and household educational level. Percentage of total natural space was significantly associated with prosocial behaviour among girls: β = 0.14 (*p* < 0.01) and among high education households: β = 0.12 (*p* < 0.05) *(significant in expected direction)*. Percentage of parks was not significantly associated with prosocial behaviour among all sub-sample groups *(non-significant in expected direction)*. Access to private garden was not significantly associated with prosocial behaviour among all sub-sample groups *(non-significant in unexpected direction)*.	Good
Sobko et al. (2018), Hong Kong	Cross-sectional study	299 (2–5 years)	Connectedness to nature (enjoyment of, empathy for, responsibility toward, and awareness of nature)	Questionnaire	Parent-reported prosocial scale from SDQ (*a continuous variable)*	No confounders adjusted in the analysis	Structural equation modelling	Greater responsibility toward nature was significantly associated with improved prosocial behaviour: β = 0.77 *(significant in expected direction)*.	Poor (no control for confounders)
Van Aart et al. (2018), Belgium	Longitudinal study	172 (6–12 years at baseline, 9–15 years at follow-up)	a. Percentage of semi-natural and forested area in a buffer of 2,000 m b. Percentage of agricultural area in a buffer of 300 m	Land cover map	Parent-reported prosocial scale from SDQ (*a continuous variable)*	Child's age, sex, and parental socio-economic status	Linear regression	Percentage semi-natural and forested area was not associated with prosocial behaviour *(non-significant in unexpected direction)*. Percentage of agricultural area was not associated with prosocial behaviour *(non-significant in expected direction)*.	Fair
van Dijk-Wesselius et al. (2018), Netherlands	Experimental study (two- group pretest-post-test design)	About 700 (7–11 years)	The intervention was carried out by increasing the presence of natural components (e.g., grass, trees) and also the quality of schoolyards (e.g., aesthetics; facilities).	In-person observation	a. Prosocial orientation assessed by self-administrated Social Orientation Choice Card (SOCC) (*a binary variable)* b. Self-reported prosocial scale from SDQ (*a continuous variable)*	Child's sex, grade level	Multi-level analysis	Analysis was stratified by grade levels (4, 5, and 6). Proportion of prosocial orientation in grades 4 and 5 in intervention compared to control group increased from baseline to the follow-up, but there was a significant decrease in grade 6 *(significant in expected and unexpected directions)*. There was no significant increase of self-reported prosocial behaviour *(non-significant in non-reported direction)*.	Fair
Whitten et al. (2018), Australia	Cross-sectional study	26,848 (mean age = 11.92 years)	Connectedness to nature	Questionnaire (self-report)	Self-reported prosocial scale from SDQ (*a continuous variable)*	Child's sex, social supports, empathy, attention, and neighbourhood socio-economic status	Linear regression	Increased connection to the nature was associated with higher prosocial behaviour: β = 0.12 (*p* < 0.001) *(significant in expected direction)*.	Fair

### Data Analysis

Quality and risk of bias of the articles were assessed using the quality assessment tools developed by the National Institutes of Health (2019) for observational and experimental studies. Similar to the process of article screening and data extraction, two reviewers independently performed the quality assessment and any discrepancies were discussed with the third reviewer. The extracted data from all eligible articles were summarised along with study quality assessment outcomes, followed by the narrative synthesis of the evidence on direction, magnitude, effect modifiers, and mediators of the association. The findings were then discussed and future study directions were proposed.

## Results

### Literature Search Results

**Figure 2:**
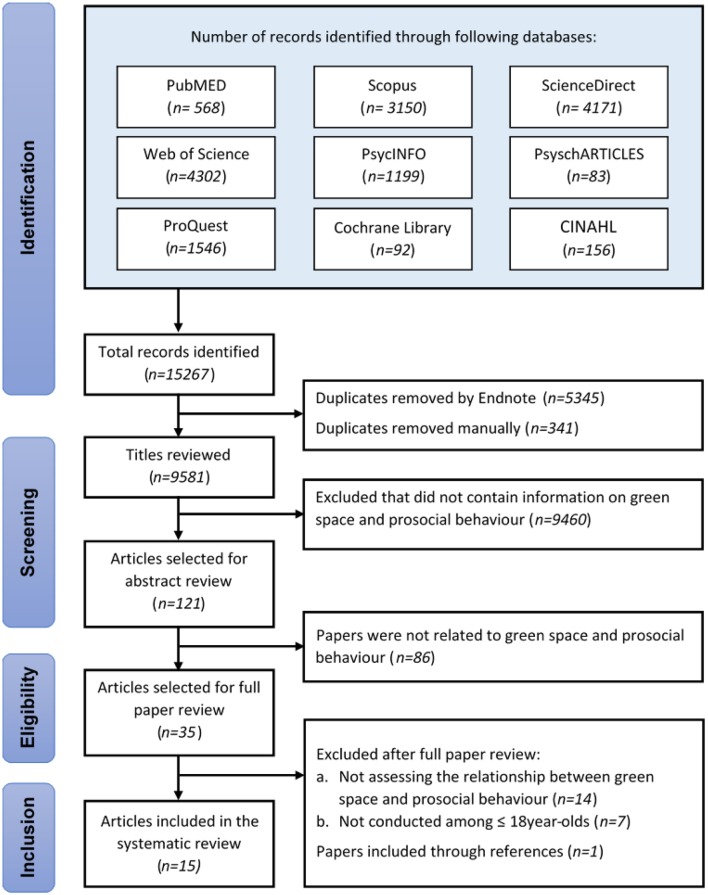
Study selection process based on PRISMA guidelines.

Figure 2 presents the search results based on the PRISMA guidelines. Out of 15,267 articles retrieved from nine databases, 5,686 duplicates were removed. Screening based on title and abstract resulted in the selection of 35 articles for the full review. After the full-text assessment, 14 studies met the eligibility criteria. During this process, one paper (Carrus et al., 2015) was identified through references, resulting in a total of 15 papers for review.

### Study Characteristics and Methods

Table 2 presents a summary for studies included in this review. All studies were from high-income countries. The majority were carried out in European countries (9; 60%), and followed by the US (3; 20%). Even though there was no restriction for publication date applied, all eligible studies were published between 2012–2019 and more than half (66.7%) were published in the last 3 years (2017–2019). There was an equal number (six studies) of cross-sectional (Odgers et al., 2012; Amoly et al., 2014; Balseviciene et al., 2014; Sobko et al., 2018; Whitten et al., 2018; Andrusaityte et al., 2019) and experimental studies (Carrus et al., 2015; Park et al., 2016; Mayfield et al., 2017; Bates et al., 2018; van Dijk-Wesselius et al., 2018; Dopko et al., 2019). The remaining studies were of a longitudinal design (Richardson et al., 2017; McEachan et al., 2018; Van Aart et al., 2018). The design of experimental studies varied with regards to the inclusion of a control group and measurement of the outcome before the intervention (pre-test). Out of two single group experimental studies, one study was a single group post-test only experiment (Bates et al., 2018), whereas another used a single group pre-post design (Park et al., 2016). The other four experimental studies reported using a control group, including two studies with—(Mayfield et al., 2017; van Dijk-Wesselius et al., 2018) and two without pre-test (Carrus et al., 2015; Dopko et al., 2019), respectively. Moreover, two (Richardson et al., 2017; McEachan et al., 2018), eight (Amoly et al., 2014; Balseviciene et al., 2014; Park et al., 2016; Mayfield et al., 2017; Van Aart et al., 2018; van Dijk-Wesselius et al., 2018; Whitten et al., 2018; Andrusaityte et al., 2019), and five (Odgers et al., 2012; Carrus et al., 2015; Bates et al., 2018; Sobko et al., 2018; Dopko et al., 2019) studies included in this review were judged to be of good, fair, and poor quality, respectively.

Sample size and age of participants differed by included study. Small sample sizes (< 100) were reported in two experimental studies (Carrus et al., 2015; Dopko et al., 2019), whilst the largest sample size was observed in a cross-sectional study of 26,848 Australian children aged 11.9 years on average (Whitten et al., 2018). Two experimental studies recorded the number of person-observations as the unit of analysis instead of number of participants (Mayfield et al., 2017; Bates et al., 2018). Furthermore, age of participants differed across studies. One of the longitudinal studies collected the baseline data of exposure during pregnancy and then did the follow-up measurement of prosocial behaviour when children were aged 4 years old (McEachan et al., 2018). In cross-sectional studies, the age of participants ranged from 2 to 12 years-old (Odgers et al., 2012; Amoly et al., 2014; Balseviciene et al., 2014; Sobko et al., 2018; Whitten et al., 2018; Andrusaityte et al., 2019). Two experimental studies did not explicitly mention the age of participants (Mayfield et al., 2017; Bates et al., 2018). The youngest participants in experimental studies were aged 1.5 years, while 8th-grade students (aged 13–14 years depending on the country) were the oldest participant.

### Green Space Measures

Green space measurements varied by study. Secondary data linked with objective measurements of area-level green space were used in seven observational studies mostly reported from European countries (Odgers et al., 2012; Amoly et al., 2014; Balseviciene et al., 2014; Richardson et al., 2017; McEachan et al., 2018; Van Aart et al., 2018; Andrusaityte et al., 2019). Green space quantity, such as residential nearby greenness, as well as the percentage of green space or other related characteristics (e.g., park space, semi-natural and forested, agricultural area) within specified distances from participants' homes were commonly used objective measurements of green space exposure. Only one study reported measuring school and combined home-school greenness in relation to prosocial behaviour (Amoly et al., 2014). In addition, residential proximity (e.g., distance to major or nearby green space) was assessed by two studies (Amoly et al., 2014; Balseviciene et al., 2014). Normalised Difference Vegetation Index (NDVI) was predominantly utilised (Amoly et al., 2014; Balseviciene et al., 2014; McEachan et al., 2018; Andrusaityte et al., 2019), followed by land cover map (Richardson et al., 2017; Van Aart et al., 2018), and Google Street View (Odgers et al., 2012).

Some studies (Amoly et al., 2014; Richardson et al., 2017; McEachan et al., 2018; Sobko et al., 2018; Whitten et al., 2018; Andrusaityte et al., 2019) also introduced subjective measures of green space and mostly relied on questionnaire-based parental-led approach. The indicator of children's time spent in green space was reported by three studies in Europe (Amoly et al., 2014; McEachan et al., 2018; Andrusaityte et al., 2019). Other studies from the UK also measured access to private gardens (Richardson et al., 2017) and satisfaction with green space (McEachan et al., 2018). Only two studies measured the contacts of green space as a perception of connectedness to nature, of which one measured connection to nature in general (Whitten et al., 2018) and the other (Sobko et al., 2018) employed multiple indicators (enjoyment of, empathy for, responsibility of, and awareness of nature).

For six experimental studies, exposure to green space was observed directly among participants. There were two main concepts of intervention model for green space exposures exhibited that included: (1) improving the appearance of frequently accessed green space by children and adolescents (e.g., schoolyards; playground markings) and (2) spending time in green space or participating in activities involving contacts with natural vegetation (e.g., horticultural programs). Improvements in the quality of schoolyards by increasing the presence of natural components and other facilities was evaluated in studies in the US (Bates et al., 2018) and the Netherlands (van Dijk-Wesselius et al., 2018), while another study in the US measured the change of prosocial behaviour due to improved playgrounds in schools (Mayfield et al., 2017). Moreover, studies in Italy (Carrus et al., 2015) and Canada (Dopko et al., 2019) compared differences in prosocial behaviour between children spending time outdoors in school green space compared to indoors within or outside a school setting. A study in South Korea observed change in prosocial behaviour after children participated in a horticultural program that facilitated contact with natural vegetation (Park et al., 2016).

### Prosocial Behaviour Measures

Even though tools for assessing prosocial behaviour varied by study, the data were mostly documented based on parental report (7; 47%). However, measurements based on teacher-reports (1; 7%), combined parent- and teacher-report (1; 7%), and self-report (2; 13%) were also observed. In addition, prosociality was assessed through in-person observations in four experimental studies (27%). The Strengths and Difficulties Questionnaire (SDQ; Goodman, 1997), which is a common tool for assessing prosocial behaviour, was employed in the majority of studies (9; 60%). This prosocial scale consists of five Likert-scale questions with a higher total score indicating more favourable prosocial behaviour. Only one study categorised a prosocial behaviour score into a binary variable using a validated cut-off point (normal with score >5; abnormal/borderline with score ≤ 5) (Andrusaityte et al., 2019). Meanwhile, experimental studies used different measures, such as the System for Observing Children's Activity and Relationship during Play (SOCARP; Mayfield et al., 2017; Bates et al., 2018), a behavioural checklist (Crust et al., 2014), assigned tasks (Dopko et al., 2019), the Social Orientation Choice Card (SOCC; van Dijk-Wesselius et al., 2018), and a questionnaire developed by previous researchers (Park et al., 2016). Three experimental studies used multiple measures of prosociality to disentangle which measure or component of prosocial behaviour is more relevant for green space exposure (Park et al., 2016; van Dijk-Wesselius et al., 2018; Dopko et al., 2019).

### Association Between Green Space and Prosocial Behaviour Among Children and Adolescents

A total of 63 associations between green space and prosocial behaviour were observed from 15 articles, including all indicators of green space and prosocial behaviour analysed within individual studies, as well as multiple analyses disaggregated by moderators (see Table 3). Exposure to green space was objectively (Odgers et al., 2012; Amoly et al., 2014; Balseviciene et al., 2014; Carrus et al., 2015; Park et al., 2016; Mayfield et al., 2017; Richardson et al., 2017; Bates et al., 2018; McEachan et al., 2018; Van Aart et al., 2018; van Dijk-Wesselius et al., 2018; Andrusaityte et al., 2019; Dopko et al., 2019) or subjectively (Amoly et al., 2014; Richardson et al., 2017; McEachan et al., 2018; Sobko et al., 2018; Whitten et al., 2018; Andrusaityte et al., 2019) measured. Overall, 44 (69.9%) out of 63 associations were in the expected direction. However, only 18 associations were reported to be statistically significant in the expected direction (Balseviciene et al., 2014; Carrus et al., 2015; Park et al., 2016; Richardson et al., 2017; Bates et al., 2018; McEachan et al., 2018; Sobko et al., 2018; van Dijk-Wesselius et al., 2018; Whitten et al., 2018; Andrusaityte et al., 2019; Dopko et al., 2019).

**Table 3:** Summary of associations extracted from 15 articles.

**Green space measurements**	***n*^**a**^**	**Association**				
		**Significant**		**Non-significant**		
		**E^**b**^**	**UE^**c**^**	**E^**b**^**	**UE^**c**^**	**NR^**d**^**
**OBJECTIVE**						
Residential surrounding greenness in buffers of:						
- 100 m	4			4		
- 250 m	1			1		
- 300 m	4		1	2	1	
- 500 m	3			3		
School greenness in a buffer of 100 m	1			1		
Home-school greenness in a buffer of 100 m	1			1		
Percentage of green or natural space in a buffer of:						
- 500 m	4	2		2	1	
- 0.5 mile (≈804.672 m)	1					
Percentage of park space in a buffer of 500 m	4			3	1	
Percentage of semi-natural and forested area in a buffer 2,000 m	1				1	
Percentage of agricultural area in a buffer 300 m	1			1		
Residential proximity to green space	3	1		2		
Schoolyard renovation^e^	7	3	1			3
Spending time in school green space^e^	5	4			1	
Playground marking^e^	4			1	3	
Participation in horticultural program^e^	4	4				
Sub-total	48	14	2	21	8	3
**SUBJECTIVE**						
Time spent in green space	4	1		2		1
Access to private garden	4				4	
Satisfaction with green space	2	1				1
Connectedness to nature	1	1				
-[-] Enjoyment of nature	1			1		
- Empathy for nature	1			1		
- Awareness of nature	1			1		
- Responsibility of nature	1	1				
Sub-total	15	4	0	5	4	2
Total: *n* (%)	63	18 (28.6)	2 (3.2)	26 (41.3)	12 (19.0)	5 (7.9)

a *Number of associations examined between green space and prosocial behaviour that count multiple indicators of green space or prosocial behaviour, as well as, multiple analyses (e.g., analysis stratified by moderators)*.

b *Association in expected direction*.

c *Association in unexpected direction*.

d *Association in non-reported direction*.

e *Green space exposures assessed by in-person observation in experimental studies*.

Two studies reported statistically significant associations between objective area-level measures of green space and prosocial behaviour after socio-demographic characteristics were counted as moderating factors (Balseviciene et al., 2014; Richardson et al., 2017). A longitudinal study in the UK reported statistically significant confounder-adjusted associations between percentage of green space in a buffer of 500 m and prosocial behaviour among 2,909 children (Richardson et al., 2017). Analyses stratified by the child' sex (males vs. females = 51 vs. 49%) and household educational level (high vs. low = 38 vs. 62%) showed that positive associations was only found among samples of girls and participants in highly educated households (Richardson et al., 2017). By contrast, a cross-sectional study in Lithuania found that increased residential greenness within a distance of 300 m from home was associated with lower levels of prosocial behaviour among children from high-educated mothers (Balseviciene et al., 2014). This study also reported an expected direction association that lower distance to city parks increased prosocial behaviour among children from low-educated mothers.

In-person observations used to measure green space exposure in experimental studies tended to report statistically significant findings. Children and adolescents who had used the quality-improved schoolyards (Bates et al., 2018; van Dijk-Wesselius et al., 2018) or participated in activities involving contact with nature (Carrus et al., 2015; Park et al., 2016; Dopko et al., 2019) had higher prosociality. One study in the Netherlands suggested that grade levels as a proxy of children's age modified the effects of intervention (van Dijk-Wesselius et al., 2018). The effects of a schoolyard renovation on child prosocial orientation varied by grade level. Among younger students (grade 4 and 5), the proportion of prosocial orientation increased from baseline to the follow-up, but a negative association was observed among older students (grade 6).

Nine out of 15 associations between subjective measures of green space and prosociality were reported in positive direction, of which only four were statistically significant. One study reported that increased time spent in city parks by 1 h per week was associated with decreased odds of borderline or abnormal prosocial behaviour after controlling for covariates (Andrusaityte et al., 2019). By contrast, studies that measured either spending time in green space as annual total hours during the last school period and holidays (Amoly et al., 2014), or time spent playing outside (minutes per week during summer and winter months; McEachan et al., 2018) did not report statistically significant associations. Only one study from Bradford, UK assessed the green space quality by asking parents about their satisfaction with frequently visited green space (McEachan et al., 2018). Analysis was disaggregated by the child's ethnicity (white British vs. south Asian), which was defined by parental report of which ethnicity they belonged to. This study found a statistically significant positive association for south Asian children, but the direction of the non-significant association was not reported among white British children. In addition, analyses of the access to private green space stratified by child's sex (male vs. female) and household educational level (low vs. high) consistently found non-significant negative associations for all sub-group analyses (Richardson et al., 2017). Furthermore, studies in Australia (Whitten et al., 2018) and Hong Kong (Sobko et al., 2018) reported that increased feelings of connection to nature and responsibility for nature were statistically significant associated with greater prosocial behaviour, respectively.

## Discussion

This review aimed to provide an overview of existing evidence assessing potential links between green space and prosocial behaviour among children and adolescents. The balance of evidence suggests that the development of prosocial behaviour may be associated with exposure to higher levels of nearby green space. However, the quality of this evidence is not yet sufficient to draw firm conclusions around causality or to offer specific guidance around well-defined interventions. Moreover, potential effect modifiers of the relationship between green space and prosocial behaviour were evident in some study contexts. Plausible mechanisms linking green space to prosociality have not been explored so far that need further investigation.

### Inconsistent Findings

Differences in methodological approaches, such as the measurement of green space, could have led to inconsistent findings. Measures of exposure to green space from included studies consisted of land cover-based metrics, distance to green space, and in-person observations, as well as subjective measurements of green space-related satisfaction, the amount of time spent outdoors, access to private gardens, and perceived connectedness to nature. There were 20 associations between green space quantity and prosocial behaviour in the expected direction, but only two associations were statistically significant. Meanwhile, five associations were reported in unexpected direction, of which one association was statistically significant. The small number of statistically significant associations in expected direction might be due to limitations in measurements. Specifically, NDVI as the common measure for area-level green space has some limitations, such as its inability to distinguish different types of green space (park, garden, etc.) and does not take into account the quality of green space including abandoned or unsafe areas (Villeneuve et al., 2018). Previous studies reported that parental concern on children's safety for playing outdoors might discourage green space use (Strife and Downey, 2009; Sefcik et al., 2019). Therefore, adequate quantity of neighbourhood green space available might not fully lead to its utilisation due to other characteristics are paid attention for children's use, such as green space quality.

Parental report on green space-related satisfaction measured in a study in Bradford, UK (McEachan et al., 2018) could be considered as a proxy of green space quality. While the higher parental satisfaction with green space was associated with greater prosocial behaviour among south Asian children, none of the green space quantity indicators was identified as a predictor of prosociality. Since children are reliant on their parents to chaperon them to green spaces, parental perceptions whether the aspects of green space quality (e.g., safety, physically attractive, etc.) meet their acceptable level might be a more reliable measurement for children's access to and use of green space. It can be an important factor for children's contact with green space than the amount of neighbourhood green space (Feng and Astell-Burt, 2017d). Three studies on child health in Australia confirmed that favourable green space quality—defined subjectively by asking parents to what extent they agreed that good parks, playgrounds, and play spaces were available in the neighbourhood—was associated with higher child well-being (Feng and Astell-Burt, 2017c,d) and general health (Feng and Astell-Burt, 2017a) independently of the green space quantity. One of those studies also reported that green space quality was a stronger determinant of children's externalising behaviours (conduct and hyperactive problems), as measured by the SDQ, than green space quantity (Feng and Astell-Burt, 2017c). It might suggest that parental report on green space quality matters in evaluating the relationship between green space and child health-related outcomes.

Out of three studies from Spain, Lithuania, UK assessing children's time spent in green space, studies that expressed time as annual total hours during the last school period and holidays in Spain (Amoly et al., 2014) and total minutes per week in summer and winter months in the UK (McEachan et al., 2018) might be prone to recall bias, leading to non-significant associations with prosocial behaviour. Meanwhile, having access to a private garden was negatively associated with prosociality in Scotland, UK, which may be because private gardens might promote less social interaction compared to public green space (Richardson et al., 2017). In addition, the use of different measurements (Connectedness to Nature Index for Parents of Preschool Children vs. combined Connection to Nature Index and Connectedness to Nature Scale) and to whom perceived connection to nature (parental report vs. self-report) was asked might generate different findings between studies in Hong Kong (Sobko et al., 2018) and Australia (Whitten et al., 2018).

The statistically significant associations between green space and prosocial behaviour were more apparent in experimental studies, which might be due to assessments of green space exposure. The more consistent association in experimental studies could be possibly due to the use of in-person observation. While cross-sectional and longitudinal studies commonly used area-level of, proximity to green space, or other subjective measurements as proxies of green space exposure, in-person observation in experimental was potentially a more accurate assessment of use and direct contact with green space among children. Indeed, having direct contact with green space may enable children to gain necessary benefits for prosocial development.

### Moderators and Mediators of the Association

Findings from the studies in this review indicating that socio-demographic background moderates associations between green space and prosocial behaviour might suggest that green space inequalities exist in some settings. For example, ethnic background was found to moderate the association between green space-related satisfaction and prosociality among chidren in Bradford, UK (McEachan et al., 2018). Within the study context in Bradford, south Asian families were found with less green space quantity and they reported less time spent in green space by their children and lower green space-related satisfaction compared to those from white British communities. A study in Kaunas, Lithuania reported an association in the non-hypothesised direction among children whose mothers had high education (Balseviciene et al., 2014). High socio-economic families in Kaunas live in suburban areas (more expensive than residing in cities) with an adequate amount of residential greenness available, but it does not promote outdoor activities due to parental concern of children's safety. Inversely, in Scotland, UK, a positive association was observed among children from high-education households (Richardson et al., 2017). These families had more green space available in their neighbourhoods, where a lack of safety might be less of an issue. In addition, this study also found a statistically significant association between green space measured as total natural space and prosocial behaviour among girls only. The characteristics of natural spaces (e.g., amenity areas, playing fields) might be more important for mentally-stimulating play and prosocial development among girls (Richardson et al., 2017). Furthermore, a moderation effect of grade level (as proxy for children's age) may indicate short-term increase in prosocial behaviour among younger, but negative impact on older children (van Dijk-Wesselius et al., 2018). To conclude, depending on the study settings, moderating variables may work in different ways.

The conceptual model described earlier suggests different pathways linking green space to child prosocial behaviour. Unfortunately, none of the included studies analysed potential mediators to test plausible linking pathways. Current literature indicates that mediators may influence this association. A study conducted among adult samples by Zhang et al. (2014) confirmed that mental health and well-being aspects (e.g., positive emotions) mediated the association between green space exposure and prosocial behaviour. In addition, Chen et al. (2019) reported bidirectional relationships between subjective well-being and prosocial behaviour among elementary school-aged children, of which, well-being leads to greater prosociality. Given the well-established relationships between green space and child mental well-being (Flouri et al., 2014; Feng and Astell-Burt, 2017c,d; McCormick, 2017; Vanaken and Danckaerts, 2018), it is plausible that mental health may mediate the association between green space and prosocial behaviour. Moreover, physical activity may also influence the green space-prosociality relationship. Recent growing literature suggest that exposure to local greenness improved physical activity among children (Roemmich et al., 2006; Sanders et al., 2015; Akpinar, 2017). Physical activity performed with other children can encourage social interactions and promote prosocial behaviour. Studies among Peruvian (Pawlowski et al., 2016) and Dutch children (Moeijes et al., 2018) confirmed that participation in a sport group fostered prosocial behaviour. A systematic review among the general population also showed that outdoor sports, in particular, can help increase prosocial behaviour (Eigenschenk et al., 2019). Therefore, child mental health and physical activity may potentially explain the relationship between green space and prosocial behaviour that needs further investigation.

In general, this review summarises preliminary evidence on the positive association between green space exposure and prosocial behaviour with some reported potential effect modifiers. However, the current available evidence available is not sufficient to infer causal associations. The longitudinal studies had short periods of observation (2–4 years) and did not account for time-variant measures of green space and prosocial behaviour. This prevents the examination of possible variations in prosocial behaviour as a response to changes in green space exposure over time. According to the conceptual framework, the accumulation of exposure to green space might elevate the benefits for prosocial behaviour development and greater impact may be observed during the late childhood as the sensitive period. Therefore, testing this hypothesis in longitudinal studies will provide new insights that will be beneficial for policy recommendations. In addition, mediation analyses are needed to test mechanistic pathways that may underlie the documented associations between green space and prosocial behaviour.

### Strengths and Limitations

To our knowledge, this is the first systematic review evaluating the relationship between green space and prosocial behaviour. The findings are presented and discussed by different measures of green space exposure with additional explanations on potential moderators. The use of nine databases with keywords adopted from current published systematic reviews, no restriction on publication date, and screening of references of included studies allowed a comprehensive search. The process of developing and reporting this review following the PRISMA guidelines lends credibility to the findings.

There are some limitations of the evidence reviewed and review method. Firstly, there was only a limited number of longitudinal studies which preclude drawing causal inferences. The findings from experimental studies without control groups are also prone to low internal validity. Secondly, area-level measures of green space varied by study and resulted in mixed-findings, making it difficult to define absolute amount of green space needed in the neighbourhood for positive development of prosocial behaviour. Thirdly, all studies were from high-income countries. Thus, findings can be applicable to these countries, including high-income countries with hot climates and rapidly growing populations where the presence of green space is substantial for mitigating harmful environmental stressors (e.g., heat) and bridging people to the community (e.g., social interactions). However, findings may not be widely applicable to middle- and low-income countries. A limitation of the review method is that some articles that were not published in English may not have been retrieved.

### Future Research Directions

This review provides preliminary evidence of positive associations between green space exposure and prosociality. However, experimental studies are just as limited as observational studies, the exposure to green space can be randomly assigned, but individual compliance in reality is agentic. Therefore, it might lead to the question of what aspects or characteristics of green space might further influence the use of green space. It is conceivable that individuals might not use green space if it is not well-maintained, physically attractive, or generally of poor quality. Therefore, the quality of green space might be an important aspect that should be considered in understanding the potential benefits of green space on human health.

Green space quality has been associated with health outcomes independently of the green space quantity (van Dillen et al., 2012). In addition, green space quality was identified to be more strongly associated with mental health outcomes than green space quantity (Francis et al., 2012; de Vries et al., 2013; Feng and Astell-Burt, 2018). Comparing between objective and subjective measurements of quality, expert-determined quality of green space involving audit tools or checklist, physical observation, GIS analyses often do not take into account the appraisal of laypeople (e.g., residents) of their environment. Laypeople are more likely to know about their environment and more qualified to assess the green space quality (Hur et al., 2010). Since they have day-to-day experiences and live in the neighbourhood, their perceptions of nearby green space are likely to be consequential for successful policymaking. The importance of subjective quality compared to objective quality of green space was noted by a study in the Netherlands (Zhang et al., 2017). This study found that subjective quality mediated the association between objective quality of green space and neighbourhood satisfaction. It strongly indicates that the perceived quality of green space was a proximate determinant for neighbourhood satisfaction and might apply to other outcomes, such as prosocial behaviour. Green space quality might be an important determinant for further study in relation to prosocial behaviour since low evidence was found on green space quantity and green space quality is less studied in relation to prosociality.

New studies with greater methodological rigor (e.g., longitudinal studies that examine time-variant measures of green space quality and prosocial behaviour for change-on-change analyses) are required to edge closer to causal inferences and evidence-based policy recommendations. Based on a conceptual model described above, using a longitudinal approach may also help to understand to what extent the accumulation of green space exposure affects the levels of prosocial behaviour in different stages of development, particularly during critical and sensitive periods of the green space-prosociality association. Assessment of potential mediators could help to test plausible pathways linking green space with prosocial behaviour. Moreover, measuring green space exposure as perceived quality is needed due to a sensitive measurement in relation to child health and behaviour outcomes. Lastly, given reported effect modifiers from previous studies, analysis of green space and prosocial behaviour should be tested across strata of other variables (e.g., socio-economic status).

## Conclusions

The current evidence shows that exposure to higher levels of green space may be associated with greater prosocial behaviour. Different measurements of green space exposure led to mixed findings. Area-level green space measures were less consistent in demonstrating statistically significant associations between green space and prosocial behaviour, whereas associations were more consistent when green space was measured using in-person observation. The number of studies was too few to draw conclusions on subjective green space measurements. Further investigation on the association between green space and prosociality is warranted, especially with studies employing longitudinal designs to confirm temporality and sensitive period, as well as, capable of testing potential effect modifiers, mediators, and measures of green space quality.

## Author Contributions

IP, TA-B, and XF conceptualised the review. IP conducted the systematic search, study quality assessment, summarised the findings, wrote, and revised the manuscript. EJ peer-reviewed the systematic search, performed full-paper assessment of the eligible articles, and reviewed the manuscript draft. TA-B, DC, SV, and XF provided critical inputs throughout the process and edited the manuscript. All authors approved the final version of the manuscript.

## Conflict of Interest

The authors declare that the research was conducted in the absence of any commercial or financial relationships that could be construed as a potential conflict of interest.

## References

[B1] Abdi B. (2010). Gender differences in social skills, problem behaviours and academic competence of Iranian kindergarten children based on their parent and teacher ratings. Proc. Soc. Behav. Sci. 5, 1175–1179. 10.1016/j.sbspro.2010.07.256

[B2] Abrams D. Van de Vyver J. Pelletier J. Cameron L. (2015). Children's prosocial behavioural intentions towards outgroup members. Br. J. Dev. Psychol. 33, 277–294. 10.1111/bjdp.12085 25773274

[B3] Aknin L. B. Broesch T. Hamlin J. K. Van de Vondervoort J. W. (2015). Prosocial behavior leads to happiness in a small-scale rural society. J. Exp. Psychol. 144, 788–795. 10.1037/xge0000082 26030168

[B4] Aknin L. B. Hamlin J. K. Dunn E. W. (2012). Giving leads to happiness in young children. PloS ONE 7:e39211. 10.1371/journal.pone.0039211 22720078 PMC3375233

[B5] Akpinar A. (2017). Urban green spaces for children: a cross-sectional study of associations with distance, physical activity, screen time, general health, and overweight. Urban Forest. Urban Green. 25, 66–73. 10.1016/j.ufug.2017.05.006

[B6] Allport G. W. Clark K. Pettigrew T. (1954). The Nature of Prejudice. Cambridge, MA: Addison-Wesley.

[B7] Amoly E. Dadvand P. Forns J. López-Vicente M. Basagaña X. Julvez J.. (2014). Green and blue spaces and behavioral development in barcelona schoolchildren: the BREATHE Project. Environ. Health Perspect. 122, 1351–1358. 10.1289/ehp.1408215 25204008 PMC4256702

[B8] Andrusaityte S. Grazuleviciene R. Dedele A. Balseviciene B. (2019). The effect of residential greenness and city park visiting habits on preschool children's mental and general health in lithuania: a cross-sectional study. Int. J. Hyg. Environ. Health. 223, 142–150. 10.1016/j.ijheh.2019.09.009 31564508

[B9] Aram F. Solgi E. Holden G. (2019). The role of green spaces in increasing social interactions in neighborhoods with periodic markets. Habit. Int. 84, 24–32. 10.1016/j.habitatint.2018.12.004

[B10] Balseviciene B. Sinkariova L. Grazuleviciene R. Andrusaityte S. Uzdanaviciute I. Dedele A.. (2014). Impact of residential greenness on preschool children's emotional and behavioral problems. Int. J. Environ. Res. Public Health 11, 6757–6770. 10.3390/ijerph110706757 24978880 PMC4113842

[B11] Bar-on M. E. (2000). The effects of television on child health: implications and recommendations. Arch. Dis. Child. 83, 289–292. 10.1136/adc.83.4.289 10999857 PMC1718503

[B12] Bar-Tal D. (1982). Sequential development of helping behavior: a cognitive-learning approach. Dev. Rev. 2, 101–124. 10.1016/0273-2297(82)90006-5

[B13] Bates C. R. Bohnert A. M. Gerstein D. E. (2018). Green schoolyards in low-income urban neighborhoods: natural spaces for positive youth development outcomes. Front. Psychol. 9:805. 10.3389/fpsyg.2018.00805 29887821 PMC5980974

[B14] Ben-Shlomo Y. Mishra G. Kuh D. (2014). “*Life* course epidemiology*,”* in Handbook of Epidemiology, eds W. Ahrens and I. Pigeot (New York, NY: Springer), 1521–1549.

[B15] Brownell C. A. (2013). Early development of prosocial behavior: current perspectives. Infancy 18, 1–9. 10.1111/infa.12004 25632273 PMC4306462

[B16] Caputi M. Lecce S. Pagnin A. Banerjee R. (2012). Longitudinal effects of theory of mind on later peer relations: the role of prosocial behavior. Dev. Psychol. 48, 257–270. 10.1037/a0025402 21895361

[B17] Carlo G. Mestre M. V. McGinley M. M. Samper P. Tur A. Sandman D. (2012). The interplay of emotional instability, empathy, and coping on prosocial and aggressive behaviors. Pers. Individ. Differ. 53, 675–680. 10.1016/j.paid.2012.05.022

[B18] Carlo G. Mestre M. V. Samper P. Tur A. Armenta B. E. (2010). The longitudinal relations among dimensions of parenting styles, sympathy, prosocial moral reasoning, and prosocial behaviors. Int. J. Behav. Dev. 35, 116–124. 10.1177/0165025410375921

[B19] Carrus G. Passiatore Y. Pirchio S. Scopelliti M. (2015). Contact with nature in educational settings might help cognitive functioning and promote positive social behaviour / El contacto con la naturaleza en los contextos educativos podría mejorar el funcionamiento cognitivo y fomentar el comportamiento social positivo. Psyecology 6, 191–212. 10.1080/21711976.2015.1026079

[B20] Cauley K. Tyler B. (1989). The relationship of self-concept to prosocial behavior in children. Early Child. Res. Q. 4, 51–60. 10.1016/S0885-2006(89)90064-1

[B21] Chen X. Tian L. Huebner E. S. (2019). Bidirectional relations between subjective well-being in school and prosocial behavior among elementary school-aged children: a longitudinal study. Child Youth Care Forum. 49, 77–95. 10.1007/s10566-019-09518-4

[B22] Collie R. J. Martin A. J. Roberts C. L. Nassar N. (2018). The roles of anxious and prosocial behavior in early academic performance: a population-based study examining unique and moderated effects. Learn. Individ. Differ. 62, 141–152. 10.1016/j.lindif.2018.02.004

[B23] Crust L. McKenna J. Spence J. Thomas C. Evans D. Bishop D. (2014). The effects of playground markings on the physical self-perceptions of 10–11-year-old school children. Phys. Educ. Sport Pedag. 19, 179–190. 10.1080/17408989.2012.732565

[B24] Dadvand P. Nazelle A. D. Triguero-Mas M. Schembari A. Cirach M.. (2012a). Surrounding greenness and exposure to air pollution during pregnancy: an analysis of personal monitoring data. Environ. Health Perspect. 120, 1286–1290. 10.1289/ehp.1104609 22647671 PMC3440116

[B25] Dadvand P. Nieuwenhuijsen M. J. Esnaola M. Forns J. Basagaña X. Alvarez-Pedrerol M. (2015). Green spaces and cognitive development in primary school children. Proc. Natl. Acad. Sci. U.S.A. 112, 7937–7942. 10.1073/pnas.1503402112 26080420 PMC4491800

[B26] Dadvand P. Sunyer J. Basagaña X. Ballester F. Lertxundi A. Fernández-Somoano A.. (2012b). Surrounding greenness and pregnancy outcomes in four Spanish birth cohorts. Environ. Health Perspect. 120, 1481–1487. 10.1289/ehp.1205244 22899599 PMC3491948

[B27] Davies K. Tropp L. R. Aron A. Pettigrew T. F. Wright S. C. (2011). Cross-group friendships and intergroup attitudes: a meta-analytic review. Pers. Soc. Psychol. Rev. 15, 332–351. 10.1177/1088868311411103 21844287

[B28] de Leeuw R. N. H. Kleemans M. Rozendaal E. Anschütz D. J. Buijzen M. (2015). The impact of prosocial television news on children's prosocial behavior: an experimental study in the netherlands. J. Child. Med. 9, 419–434. 10.1080/17482798.2015.1089297

[B29] de Vries S. van Dillen S. M. E. Groenewegen P. P. Spreeuwenberg P. (2013). Streetscape greenery and health: stress, social cohesion and physical activity as mediators. Soc. Sci. Med. 94, 26–33. 10.1016/j.socscimed.2013.06.030 23931942

[B30] Dennis M. James P. (2016). User participation in urban green commons: exploring the links between access, voluntarism, biodiversity and well being. Urban Forest. Urban Green. 15, 22–31. 10.1016/j.ufug.2015.11.009

[B31] Di Bartolomeo G. Papa S. (2017). The effects of physical activity on social interactions: the case of trust and trustworthiness. J. Sports Econ. 20, 50–71. 10.1177/1527002517717299

[B32] Dinnie E. Brown K. M. Morris S. (2013). Community, cooperation and conflict: negotiating the social well-being benefits of urban greenspace experiences. Landsc. Urban Plan. 112, 1–9. 10.1016/j.landurbplan.2012.12.012

[B33] Dopko R. L. Capaldi C. A. Zelenski J. M. (2019). The psychological and social benefits of a nature experience for children: a preliminary investigation. J. Environ. Psychol. 63, 134–138. 10.1016/j.jenvp.2019.05.002

[B34] Dunfield K. A. (2014). A construct divided: prosocial behavior as helping, sharing, and comforting subtypes. Front. Psychol. 5:958. 10.3389/fpsyg.2014.00958 25228893 PMC4151454

[B35] Eigenschenk B. Thomann A. McClure M. Davies L. Gregory M. Dettweiler U.. (2019). Benefits of outdoor sports for society. a systematic literature review and reflections on evidence. Int. J. Environ. Res. Public Health 16:937. 10.3390/ijerph16060937 30875938 PMC6466442

[B36] Eisenberg N. Spinrad T. L. Knafo-Noam A. (2015). “Prosocial development,” in Handbook of Child Psychology and Developmental Science, ed R. M. Lerner (John Wiley & Sons), 1−47.

[B37] Eldeirawi K. Kunzweiler C. Zenk S. Finn P. Nyenhuis S. Rosenberg N.. (2019). Associations of urban greenness with asthma and respiratory symptoms in Mexican American children. Ann. Allergy Asthma Immunol. 122, 289–295. 10.1016/j.anai.2018.12.009 30557617

[B38] Fabes R. A. Hanish L. D. Martin C. L. Moss A. Reesing A. (2012). The effects of young children's affiliations with prosocial peers on subsequent emotionality in peer interactions. Br. J. Dev. Psychol. 30, 569–585. 10.1111/j.2044-835X.2011.02073.x 23039333 PMC3466482

[B39] Feng X. Astell-Burt T. (2017a). Do greener areas promote more equitable child health? Health Place 46, 267–273. 10.1016/j.healthplace.2017.05.006 28666236

[B40] Feng X. Astell-Burt T. (2017b). Is neighborhood green space protective against associations between child asthma, neighborhood traffic volume and area safety? Multilevel analysis of 4,447 Australian Children. J. Transport. Health 5, S40–S41. 10.1016/j.jth.2017.05.328 PMC5451993 28534841

[B41] Feng X. Astell-Burt T. (2017c). The relationship between neighbourhood green space and child mental wellbeing depends upon whom you ask: multilevel evidence from 3083 children aged 12-13 years. Int. J. Environ. Res. Public Health 14:235. 10.3390/ijerph14030235 28264461 PMC5369071

[B42] Feng X. Astell-Burt T. (2017d). Residential green space quantity and quality and child well-being: a longitudinal study. Am. J. Prev. Med. 53, 616–624. 10.1016/j.amepre.2017.06.035 28864128

[B43] Feng X. Astell-Burt T. (2018). Residential green space quantity and quality and symptoms of psychological distress: a 15-year longitudinal study of 3897 women in postpartum. BMC Psychiatry 18:348. 10.1186/s12888-018-1926-1 30367610 PMC6204015

[B44] Ferreira T. Cadima J. Matias M. Vieira J. M. Leal T. Matos P. M. (2016). Preschool Children's prosocial behavior: the role of mother–child, father–child and teacher–child relationships. J. Child Family Stud. 25, 1829–1839. 10.1007/s10826-016-0369-x

[B45] Flouri E. Midouhas E. Joshi H. (2014). The role of urban neighbourhood green space in children's emotional and behavioural resilience. J. Environ. Psychol. 40, 179–186. 10.1016/j.jenvp.2014.06.007

[B46] Flouri E. Sarmadi Z. (2016). Prosocial behavior and childhood trajectories of internalizing and externalizing problems: the role of neighborhood and school contexts. Dev. Psychol. 52, 253–258. 10.1037/dev0000076 26619321 PMC4725335

[B47] Flynn E. Ehrenreich S. E. Beron K. J. Underwood M. K. (2015). Prosocial behavior: long-term trajectories and psychosocial outcomes. Soc. Dev. 24, 462–482. 10.1111/sode.12100 26236108 PMC4517683

[B48] Fortuna K. Knafo A. (2014). “Parental and genetic contributions to prosocial behavior during childhood,” in Prosocial Development: A Multidimensional Approach, eds L. M. Padilla-Walker and G. Carlo (New York, NY: Oxford University Press), 70–89.

[B49] Francis J. Wood L. J. Knuiman M. Giles-Corti B. (2012). Quality or quantity? Exploring the relationship between public open space attributes and mental health in perth, western Australia. Soc. Sci. Med. 74, 1570–1577. 10.1016/j.socscimed.2012.01.032 22464220

[B50] Fujisawa K. K. Kutsukake N. Hasegawa T. (2008). Reciprocity of prosocial behavior in Japanese preschool children. Int. J. Behav. Dev. 32, 89–97. 10.1177/0165025407084055

[B51] Gallitto E. Leth-Steensen C. (2019). Moderating effect of trait emotional intelligence on the relationship between parental nurturance and prosocial behaviour. J. Adolesc. 74, 113–119. 10.1016/j.adolescence.2019.04.008 31195234

[B52] Garaigordobil M. (2009). A comparative analysis of empathy in childhood and adolescence: gender differences and associated socio-emotional variables. Int. J. Psychol. Psychol. Ther. 9, 217–235. Available online at: https://psycnet.apa.org/record/2009-09327-006

[B53] Gerbino M. Zuffiano A. Eisenberg N. Castellani V. Luengo Kanacri B. P. Pastorelli C.. (2018). Adolescents' prosocial behavior predicts good grades beyond intelligence and personality traits. J. Pers. 86, 247–260. 10.1111/jopy.12309 28236293

[B54] Goldy S. P. Piff P. K. (2020). Toward a social ecology of prosociality: why, when, and where nature enhances social connection. Curr. Opin. Psychol. 32, 27–31. 10.1016/j.copsyc.2019.06.016 31362182

[B55] Goodman R. (1997). The strengths and difficulties questionnaire: a research note. J. Child Psychol. Psychiatry 38, 581–586. 9255702 10.1111/j.1469-7610.1997.tb01545.x

[B56] Greitemeyer T. (2011). Effects of prosocial media on social behavior:when and why does media exposure affect helping and aggression? Curr. Direct. Psychol. Sci. 20, 251–255. 10.1177/0963721411415229

[B57] Gustin K. Tofail F. Vahter M. Kippler M. (2018). Cadmium exposure and cognitive abilities and behavior at 10 years of age: a prospective cohort study. Environ. Int. 113, 259–268. 10.1016/j.envint.2018.02.020 29459184

[B58] Hammond S. I. Waugh W. Satlof-Bedrick E. Brownell C. A. (2015). “Prosocial behavior during childhood and cultural variations,” in International Encyclopedia of the Social and Behavioral Sciences, 2nd Edn, ed J. D. Wright (Oxford: Elsevier), 228–232.

[B59] Hartig T. Mitchell R. Vries S.d Frumkin H. (2014). Nature and health. Annu. Rev. Public Health 35, 207–228. 10.1146/annurev-publhealth-032013-182443 24387090

[B60] Hay D. F. (1994). Prosocial development. J. Child Psychol. Psychiatry 35, 29–71. 10.1111/j.1469-7610.1994.tb01132.x 8163628

[B61] Hay D. F. Cook K. V. (2007). “The transformation of prosocial behavior from infancy to childhood,” in Socioemotional Development in the Toddler Years: Transitions and Transformations, eds. C. A. Brownell and C. B. Kopp (New York, NY: Guilford Press), 100–131.

[B62] Hay D. F. Payne A. Chadwick A. (2004). Peer relations in childhood. J. Child Psychol. Psychiatry 45, 84–108. 10.1046/j.0021-9630.2003.00308.x 14959804

[B63] Healy S. Garcia J. M. (2019). Psychosocial correlates of physical activity participation and screen-time in typically developing children and children on the autism spectrum. J. Dev. Phys. Disab. 31, 313–328. 10.1007/s10882-018-9642-9

[B64] Hong A. Sallis J. F. King A. C. Conway T. L. Saelens B. Cain K. L.. (2018). Linking green space to neighborhood social capital in older adults: the role of perceived safety. Soc. Sci. Med. 207, 38–45. 10.1016/j.socscimed.2018.04.051 29727748

[B65] Houlden V. Weich S. Porto de Albuquerque J. Jarvis S. Rees K. (2018). The relationship between greenspace and the mental wellbeing of adults: a systematic review. PLoS ONE 13:e0203000. 10.1371/journal.pone.0203000 30208073 PMC6135392

[B66] Hur M. Nasar J. L. Chun B. (2010). Neighborhood satisfaction, physical and perceived naturalness and openness. J. Environ. Psychol. 30, 52–59. 10.1016/j.jenvp.2009.05.005

[B67] Israel S. Hasenfratz L. Knafo-Noam A. (2015). The genetics of morality and prosociality. Curr. Opin. Psychol. 6, 55–59. 10.1016/j.copsyc.2015.03.027

[B68] Jennings V. Bamkole O. (2019). The relationship between social cohesion and urban green space: an avenue for health promotion. Int. J. Environ. Res. Public Health 16:452. 10.3390/ijerph16030452 30720732 PMC6388234

[B69] Kaplan S. (1995). The restorative benefits of nature: toward an integrative framework. J. Environ. Psychol. 15, 169–182. 10.1016/0272-4944(95)90001-2

[B70] Kazmierczak A. (2013). The contribution of local parks to neighbourhood social ties. Landsc. Urban Plan. 109, 31–44. 10.1016/j.landurbplan.2012.05.007

[B71] Knafo A. Zahn-Waxler C. Van Hulle C. Robinson J. L. Rhee S. H. (2008). The developmental origins of a disposition toward empathy: genetic and environmental contributions. Emotion 8, 737–752. 10.1037/a0014179 19102585

[B72] Knafo-Noam A. Uzefovsky F. Israel S. Davidov M. Zahn-Waxler C. (2015). The prosocial personality and its facets: genetic and environmental architecture of mother-reported behavior of 7-year-old twins. Front. Psychol. 6:112. 10.3389/fpsyg.2015.00112 25762952 PMC4327421

[B73] Kok R. Prinzie P. Bakermans-Kranenburg M. J. Verhulst F. C. White T. Tiemeier H.. (2018). Socialization of prosocial behavior: gender differences in the mediating role of child brain volume. Child Neuropsychol. 24, 723–733. 10.1080/09297049.2017.1338340 28627295

[B74] Kondo M. C. Fluehr J. M. McKeon T. Branas C. C. (2018). Urban green space and its impact on human health. Int. J. Environ. Res. Public Health 15:445. 10.3390/ijerph15030445 29510520 PMC5876990

[B75] Kuh D. Ben-Shlomo Y. Lynch J. Hallqvist J. Power C. (2003). Life course epidemiology. J. Epidemiol. Commun. Health 57, 778–783. 10.1136/jech.57.10.778 14573579 PMC1732305

[B76] Lai F. H Siu A. M Shek D. T. (2015). Individual and social predictors of prosocial behavior among Chinese adolescents in Hong Kong. Front. Pediatr. 3:39. 10.3389/fped.2015.00039 26029684 PMC4432674

[B77] Lee A. C. Maheswaran R. (2011). The health benefits of urban green spaces: a review of the evidence. J. Public Health 33, 212–222. 10.1093/pubmed/fdq068 20833671

[B78] Lee C.-T. Padilla-Walker L. M. Memmott-Elison M. K. (2016). The role of parents and peers on adolescents' prosocial behavior and substance use. J. Soc. Pers. Relat. 34, 1053–1069. 10.1177/0265407516665928

[B79] Leventhal K. S. Gillham J. DeMaria L. Andrew G. Peabody J. Leventhal S. (2015). Building psychosocial assets and wellbeing among adolescent girls: a randomized controlled trial. J. Adolesc. 45, 284–295. 10.1016/j.adolescence.2015.09.011 26547145

[B80] Liao J. Zhang B. Xia W. Cao Z. Zhang Y. Liang S.. (2019). Residential exposure to green space and early childhood neuro development. Environ. Int. 128, 70–76. 10.1016/j.envint.2019.03.070 31029981

[B81] Markevych I. Schoierer J. Hartig T. Chudnovsky A. Hystad P. Dzhambov A. M.. (2017). Exploring pathways linking greenspace to health: theoretical and methodological guidance. Environ. Res. 158, 301–317. 10.1016/j.envres.2017.06.028 28672128

[B82] Marselle M. R. Irvine K. N. Lorenzo-Arribas A. Warber S. L. (2014). Moving beyond green: exploring the relationship of environment type and indicators of perceived environmental quality on emotional well-being following group walks. Int. J. Environ. Res. Public Health 12, 106–130. 10.3390/ijerph120100106 25546275 PMC4306852

[B83] Mayfield C. A. Child S. Weaver R. G. Zarrett N. Beets M. W. Moore J. B. (2017). Effectiveness of a playground intervention for antisocial, prosocial, and physical activity behaviors. J. School Health 87, 338–345. 10.1111/josh.12506 28382669

[B84] McCormack G. R. Rock M. Toohey A. M. Hignell D. (2010). Characteristics of urban parks associated with park use and physical activity: a review of qualitative research. Health Place 16, 712–726. 10.1016/j.healthplace.2010.03.003 20356780

[B85] McCormick R. (2017). Does access to green space impact the mental well-being of children: a systematic review. J. Pediatr. Nurs. 37, 3–7. 10.1016/j.pedn.2017.08.027 28882650

[B86] McEachan R. R. C. Yang T. C. Roberts H. Pickett K. E. Arseneau-Powell D. Gidlow C. J.. (2018). Availability, use of, and satisfaction with green space, and children's mental wellbeing at age 4 years in a multicultural, deprived, urban area: results from the Born in Bradford cohort study. Lancet. Planet Health 2, e244–e254. 10.1016/s2542-5196(18)30119-0 29880156

[B87] Meleady R. Seger C. R. (2016). Imagined contact encourages prosocial behavior towards outgroup members. Group Process. Intergr. Relations 20, 447–464. 10.1177/1368430215612225

[B88] Moeijes J. van Busschbach J. T. Bosscher R. J. Twisk J. W. R. (2018). Sports participation and psychosocial health: a longitudinal observational study in children. BMC Public Health 18:702. 10.1186/s12889-018-5624-1 29879933 PMC5992880

[B89] Moher D. Liberati A. Tetzlaff J. Altman D. G. The P. G. (2009). Preferred reporting items for systematic reviews and meta-analyses: the PRISMA statement. PLoS Med. 6:e1000097 10.1371/journal.pmed.1000097 19621072 PMC2707599

[B90] National Institutes of Health (2019). Study Quality Assessment Tools. US Department of Health and Human Services. Available online at: https://www.nhlbi.nih.gov/health-topics/study-quality-assessment-tools (accessed September 7, 2019).

[B91] Obsuth I. Eisner M. P. Malti T. Ribeaud D. (2015). The developmental relation between aggressive behaviour and prosocial behaviour: a 5-year longitudinal study. BMC Psychol. 3:16. 10.1186/s40359-015-0073-4 26000166 PMC4440499

[B92] Odgers C. L. Caspi A. Bates C. J. Sampson R. J. Moffitt T. E. (2012). Systematic social observation of children's neighborhoods using google street view: a reliable and cost-effective method. J. Child Psychol. Psychiatry 53, 1009–1017. 10.1111/j.1469-7610.2012.02565.x 22676812 PMC3537178

[B93] Oerlemans A. M. Rommelse N. N. J. Buitelaar J. K. Hartman C. A. (2018). Examining the intertwined development of prosocial skills and ASD symptoms in adolescence. Eur. Child Adolesc. Psychiatry 27, 1033–1046. 10.1007/s00787-018-1114-3 29383553 PMC6060879

[B94] Ohly H. White M. P. Wheeler B. W. Bethel A. Ukoumunne O. C. Nikolaou V.. (2016). Attention restoration theory: a systematic review of the attention restoration potential of exposure to natural environments. J. Toxicol. Environ. Health Part B 19, 305–343. 10.1080/10937404.2016.1196155 27668460

[B95] Oldfield J. Humphrey N. Hebron J. (2016). The role of parental and peer attachment relationships and school connectedness in predicting adolescent mental health outcomes. Child Adolesc. Mental Health 21, 21–29. 10.1111/camh.12108 32680365

[B96] Oviedo L. (2016). Religious attitudes and prosocial behavior: a systematic review of published research. Religion Brain Behav. 6, 169–184. 10.1080/2153599X.2014.992803

[B97] Park S. A. Cho M. K. Yoo M. H. Kim S. Y. Im E. A. Song J. E. (2016). Horticultural activity program for improving emotional intelligence, prosocial behavior, and scientific investigation abilities and attitudes in kindergarteners. Hort Technol. 26, 754–761. 10.21273/HORTTECH03489-16

[B98] Pastorelli C. Lansford J. E. Luengo Kanacri B. P. Malone P. S. Di Giunta L. Bacchini D.. (2016). Positive parenting and children's prosocial behavior in eight countries. J. Child Psychol. Psychiatry Allied Dis. 57, 824–834. 10.1111/jcpp.12477 26511201 PMC4848190

[B99] Pawlowski T. Schüttoff U. Downward P. Lechner M. (2016). Can sport really help to meet the millennium development goals? Evidence from children in Peru. J. Sports Econ. 19, 498–521. 10.1177/1527002516661601

[B100] Pettygrove D. M. Hammond S. I. Karahuta E. L. Waugh W. E. Brownell C. A. (2013). From cleaning up to helping out: parental socialization and children's early prosocial behavior. Infant. Behav. Dev. 36, 843–846. 10.1016/j.infbeh.2013.09.005 24140842

[B101] Piliavin J. (2001). “Altruism and prosocial behavior, sociology of,” in International Encyclopedia of the Social and Behavioral Sciences, eds N. J. Smelser and P. B. Baltes (Oxford: Elsevier Science Ltd). 411–415.

[B102] Piotrowski J. T. Vossen H. G. M. Valkenburg P. M. (2015). “Media and child development,” in International Encyclopedia of the Social & Behavioral Sciences, 2nd Edn, ed J. D. Wright (Oxford: Elsevier), 1–10.

[B103] Proctor C. Linley P. A. (2014). “Life satisfaction in youth,” in Increasing Psychological well-being in Clinical and Educational Settings: Interventions and Cultural Contexts, eds G. A. Fava and C. Ruini (New York, NY: Springer Science + Business Media), 199–215.

[B104] Prot S. Gentile D. A. Anderson C. A. Suzuki K. Swing E. Lim K. M.. (2014). Long-term relations among prosocial-media use, empathy, and prosocial behavior. Psychol. Sci. 25, 358–368. 10.1177/0956797613503854 24335350

[B105] Qureshi F. Koenen K. C. Tiemeier H. Williams M. A. Misra S. Kubzansky L. D. (2019). Childhood assets and cardiometabolic health in adolescence. Pediatrics 143:e20182004. 10.1542/peds.2018-2004 30718380 PMC6398368

[B106] Ren Y. Yao X. Liu Y. Liu S. Li X. Huang Q.. (2019). Outdoor air pollution pregnancy exposures are associated with behavioral problems in China's preschoolers. Environ. Sci. Pollut. Res. Int. 26, 2397–2408. 10.1007/s11356-018-3715-2 30467751

[B107] Richardson E. A. Pearce J. Shortt N. K. Mitchell R. (2017). The role of public and private natural space in children's social, emotional and behavioural development in Scotland: a longitudinal study. Environ. Res. 158, 729–736. 10.1016/j.envres.2017.07.038 28750342 PMC5571194

[B108] Richman C. L. Berry C. Bittle M. Himan K. (1988). Factors related to helping behavior in preschool-age children. J. Appl. Dev. Psychol. 9, 151–165. 10.1016/0193-3973(88)90020-2

[B109] Roemmich J. N. Epstein L. H. Raja S. Yin L. Robinson J. Winiewicz D. (2006). Association of access to parks and recreational facilities with the physical activity of young children. Prev. Med. 43, 437–441. 10.1016/j.ypmed.2006.07.007 16928396

[B110] Sanders T. Feng X. Fahey P. P. Lonsdale C. Astell-Burt T. (2015). The influence of neighbourhood green space on children's physical activity and screen time: findings from the longitudinal study of Australian children. Int. J. Behav. Nutr. Phys. Act. 12:126. 10.1186/s12966-015-0288-z 26419752 PMC4589082

[B111] Schwartz A. J. Dodds P. S. O'Neil-Dunne J. P. M. Danforth C. M. Ricketts T. H. (2019). Visitors to urban greenspace have higher sentiment and lower negativity on Twitter. People Nat. 1, 476–485. 10.1002/pan3.10045

[B112] Sefcik J. S. Kondo M. C. Klusaritz H. Sarantschin E. Solomon S. Roepke A.. (2019). Perceptions of nature and access to green space in four urban neighborhoods. Int. J. Environ. Res. Public Health 16:2313. 10.3390/ijerph16132313 31261862 PMC6651051

[B113] Silke C. Brady B. Boylan C. Dolan P. (2018). Factors influencing the development of empathy and pro-social behaviour among adolescents: a systematic review. Child. Youth Serv. Rev. 94, 421–436. 10.1016/j.childyouth.2018.07.027

[B114] Singer D. G. Singer J. L. D'Agnostino H. DeLong R. (2009). Children's pastimes and play in sixteen nations: is free-play declining? Am. J. Play 1, 283–312. Available online at: https://eric.ed.gov/?id=EJ1069041

[B115] Smith E. P. Witherspoon D. P. Bhargava S. Bermudez J. M. (2019). Cultural values and behavior among African American and European American children. J. Child Family Stud. 28, 1236–1249. 10.1007/s10826-019-01367-y 31871395 PMC6927402

[B116] Sobko T. Jia Z. Brown G. (2018). Measuring connectedness to nature in preschool children in an urban setting and its relation to psychological functioning. PLoS ONE 13:e0207057. 10.1371/journal.pone.0207057 30496300 PMC6264829

[B117] Strife S. Downey L. (2009). Childhood development and access to nature: a new direction for environmental inequality research. Organ. Environ. 22, 99–122. 10.1177/1086026609333340 21874103 PMC3162362

[B118] Su J. G. Jerrett M. de Nazelle A. Wolch J. (2011). Does exposure to air pollution in urban parks have socioeconomic, racial or ethnic gradients? Environ. Res. 111, 319–328. 10.1016/j.envres.2011.01.002 21292252

[B119] Swit C. (2012). *Relational aggression and prosocial behaviours in Australian preschool* children. Aust. J. Early. Child. 37, 30–34. 10.1177/183693911203700305

[B120] Taylor L. Hochuli D. F. (2017). Defining greenspace: multiple uses across multiple disciplines. Landsc. Urban Plan. 158, 25–38. 10.1016/j.landurbplan.2016.09.024

[B121] Tischer C. Gascon M. Fernández-Somoano A. Tardón A. Lertxundi Materola A. Ibarluzea J.. (2017). Urban green and grey space in relation to respiratory health in children. Eur. Respir. J. 49:1502112. 10.1183/13993003.02112-2015 28642307

[B122] Twohig-Bennett C. Jones A. (2018). The health benefits of the great outdoors: a systematic review and meta-analysis of greenspace exposure and health outcomes. Environ. Res. 166, 628–637. 10.1016/j.envres.2018.06.030 29982151 PMC6562165

[B123] Ulrich R. S. (1983). “Aesthetic and affective response to natural environment,” in Behavior and the Natural Environment, eds I. Altman and J. F. Wohlwill (Boston, MA: Springer US), 85–125.

[B124] Ulrich R. S. Simons R. F. Losito B. D. Fiorito E. Miles M. A. Zelson M. (1991). Stress recovery during exposure to natural and urban environments. J. Environ. Psychol. 11, 201–230. 10.1016/S0272-4944(05)80184-7

[B125] Van Aart C. J. C. Michels N. Sioen I. De Decker A. Bijnens E. M. Janssen B. G.. (2018). Residential landscape as a predictor of psychosocial stress in the life course from childhood to adolescence. Environ. Int. 120, 456–463. 10.1016/j.envint.2018.08.028 30145309

[B126] van den Berg M. Wendel-Vos W. van Poppel M. Kemper H. van Mechelen W. Maas J. (2015). Health benefits of green spaces in the living environment: a systematic review of epidemiological studies. Urban Forest.Urban Green. 14, 806–816. 10.1016/j.ufug.2015.07.008

[B127] van Dijk-Wesselius J. E. Maas J. Hovinga D. van Vugt M. van den Berg A. E. (2018). The impact of greening schoolyards on the appreciation, and physical, cognitive and social-emotional well-being of schoolchildren: a prospective intervention study. Landsc. Urban Plan. 180, 15–26. 10.1016/j.landurbplan.2018.08.003

[B128] van Dillen S. M. E. de Vries S. Groenewegen P. P. Spreeuwenberg P. (2012). Greenspace in urban neighbourhoods and residents' health: adding quality to quantity. J. Epidemiol. Commun. Health 66:e8. 10.1136/jech.2009.104695 21715445

[B129] Vanaken G. J. Danckaerts M. (2018). Impact of green space exposure on children's and adolescents' mental health: a systematic review. Int. J. Environ. Res. Public Health 15:2668. 10.3390/ijerph15122668 30486416 PMC6313536

[B130] Vilar M. M. Corell L. Merino C. (2019). Systematic review of prosocial behavior *measures* 37, 349–377. 10.18800/psico.201901.012

[B131] Villeneuve P. J. Ysseldyk R. L. Root A. Ambrose S. DiMuzio J. Kumar N.. (2018). Comparing the normalized difference vegetation index with the google street view measure of vegetation to assess associations between greenness, walkability, recreational physical activity, and health in Ottawa, Canada. Int. J. Environ. Res. Public Health 15:1719. 10.3390/ijerph15081719 30103456 PMC6121879

[B132] Ward J. S. Duncan J. S. Jarden A. Stewart T. (2016). The impact of children's exposure to greenspace on physical activity, cognitive development, emotional wellbeing, and ability to appraise risk. Health Place 40, 44–50. 10.1016/j.healthplace.2016.04.015 27179137

[B133] Weinstein B. D. Bearison D. J. (1985). Social interaction, social observation, and cognitive development in young children. Eur. J. Soc. Psychol. 15, 333–343.

[B134] Wentzel K. (2015). “Prosocial behaviour and schooling,” in Encyclopedia on Early Childhood Development, eds R. Tremblay, M. Boivin, and R. Peters. (The Centre of Excellence for Early Childhood Development (CEECD)). Available online at: http://www.child-encyclopedia.com/prosocial-behaviour/according-experts/prosocial-behaviour-and-schooling

[B135] Whitten T. Stevens R. Ructtinger L. Tzoumakis S. Green M. J. Laurens K. R. (2018). Connection to the natural environment and well-being in middle childhood. Ecopsychology 10, 270–279. 10.1089/eco.2018.0010

[B136] Williams A. O'Driscoll K. Moore C. (2014). The influence of empathic concern on prosocial behavior in children. Front. Psychol. 5:425. 10.3389/fpsyg.2014.00425 24860537 PMC4026684

[B137] Wittek R. Bekkers R. (2015). “Altruism and prosocial behavior, sociology of,” in International Encyclopedia of the Social & Behavioral Sciences, 2nd Edn, ed J. D. Wright (Oxford: Elsevier), 579–583.

[B138] Yang Y. Li W. Sheldon K. M. Kou Y. (2019). Chinese adolescents with higher social dominance orientation are less prosocial and less happy: a value-environment fit analysis. Int. J. Psychol. 54, 325–332. 10.1002/ijop.12474 29318618

[B139] Zhang J. W. Piff P. K. Iyer R. Koleva S. Keltner D. (2014). An occasion for unselfing: Beautiful nature leads to prosociality. J. Environ. Psychol. 37, 61–72. 10.1016/j.jenvp.2013.11.008

[B140] Zhang Y. Van den Berg A. E. Van Dijk T. Weitkamp G. (2017). Quality over quantity: contribution of urban green space to neighborhood satisfaction. Int. J. Environ. Res. Public Health 14:535. 10.3390/ijerph14050535 28509879 PMC5451986

